# Linescan microscopy data to extract diffusion coefficient of a fluorescent species using a commercial confocal microscope

**DOI:** 10.1016/j.dib.2019.105063

**Published:** 2020-01-02

**Authors:** Marc Bathe-Peters, Philipp Gmach, Paolo Annibale, Martin J. Lohse

**Affiliations:** a Max Delbrück Center for Molecular Medicine in the Helmholtz Association, Berlin, Germany; b Julius Maximilian University, Würzburg, Germany

**Keywords:** Confocal microscopy, Fluorescence correlation spectroscopy, Membrane receptor, Diffusion

## Abstract

We report here on the measurement of the diffusion coefficient of fluorescent species using a commercial microscope possessing a resonant scanner. Sequential linescans with a rate of up to 12 kHz yield a temporal resolution of 83 μs, making the setup amenable to measure diffusion rates over a range covering at least three orders of magnitude, from 100 μm^2^/s down to 0.1 μm^2^/s. We share representative data sets covering (i) the diffusion of a dye molecule, observed in media of different viscosities and (ii) the diffusion of a prototypical membrane receptor.

The data can be valuable for researchers interested in the rapid diffusion properties of nuclear, cytosolic or membrane bound proteins fused to fluorescent tags.

**Table:** Specifications Table

Subject	Biophysics
Specific subject area	Fluorescence Spectroscopy
Type of data	Table Image Graph Figure
How data were acquired	Confocal Laser Scanning Microscope Leica Sp8 WLL
Data format	Raw
Parameters for data collection	Solution of the relevant dyes were prepared at concentrations ranging from 10 to 100 nM. Water-glycerol mixtures were prepared by weight, an thoroughly mixed to achieve a homogeneous solution.
Description of data collection	Linescans were collected at up to 12 kHz, with line sizes of either 128 or 256 pixels. Pixel size was set at 50 nm. A number of lines comprised between 3 × 10^5^ and 2 × 10^6^ were collected. The IR laser based autofocus of the microscope (Leica, Adaptive Focus Control) was enabled during the acquisition to stabilise the focal position. The objective turret was held at the constant temperature of 20 °C by a cooling circuit connected to a chiller. The microscope was mounted on a pneumatic insulation table.
Data source location	Institution: Max Delbrück Center for Molecular Medicine City/Town/Region: Berlin Country: Germany
Data accessibility	Repository name: Mendeley Data Data identification number: Direct URL to data: https://data.mendeley.com/datasets/c2bdg4c7x7/draft?a=42fa87bd-650f-4f1e-8824-fddfc9f84c06

**Table:** 

**Value of the Data** • These data and the associated analysis pipeline provide a walkthrough on how to measure diffusion of any fluorescent species using sequential linescans on a commercial confocal microscope • One of our datasets, reporting the diffusion of a fluorescently labelled membrane receptor, provides a useful benchmark for researchers performing similar experiments. • These data will be of interest to all those scientists who have access to a commercial confocal microscope of the last generation, allowing line-scan rates of the order of 1 kHz or higher (depending on the rapidity of the species under investigation). • The data can be used to test and develop algorithms to extract the diffusion coefficient from the spatial and temporal fluctuations present in the data.

## 1 Data

**Fig. 1:**
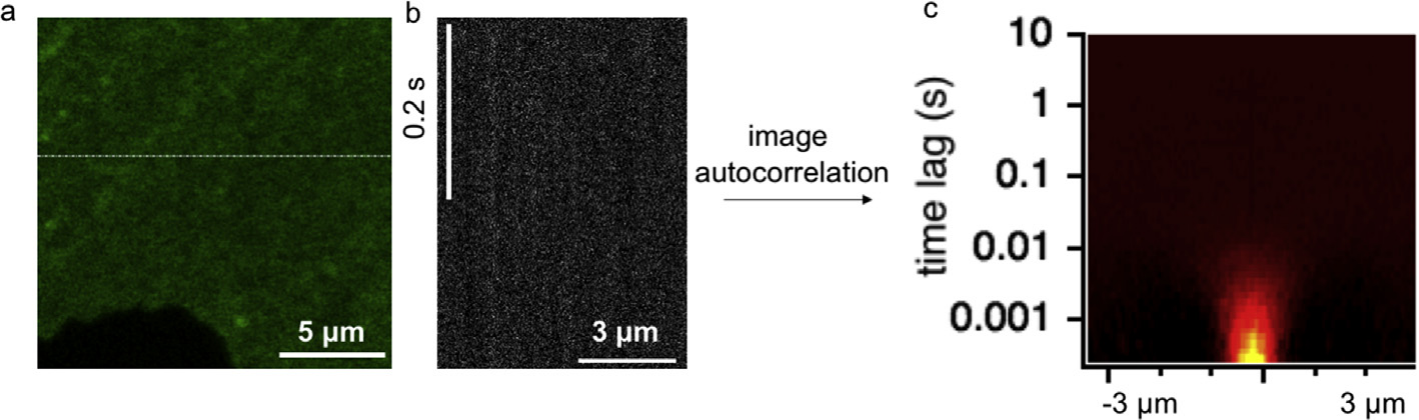
a) Representative confocal microscopy micrograph of H9c2 cell expressing a β2 Adrenergic Receptor, c-terminally fused to EGFP. b) Section of a kymograph arising from repeated linescan analysis of the line highlighted in panel a. Vertical axis represents time, horizontal axis space. c) Resulting spatial-temporal correlation function. Vertical axis represents time-lag, horizontal axis spatial distance *x* (in μm).

The shared data are kymographs (from here on linescans) collected to characterize the performance of a commercial laser scanning microscope in order to recover the diffusion coefficient of a fluorescent species. Here, the confocal beam is repeatedly scanned at high speed over the same portion of the sample. The behavior of the diffusing species can be extracted from the raw linescans by calculating the autocorrelation function. The calculation of the spatial (along the line, *x*) and temporal (along each timepoint associated to a pixel, *t*) correlation provides the Spatial Temporal Correlation Function (STICS) [[1], [2], [3]], which reflects the diffusion speed of the species under investigation. This process is illustrated in Fig. 1 and further described in the Methods.

**Fig. 2:**
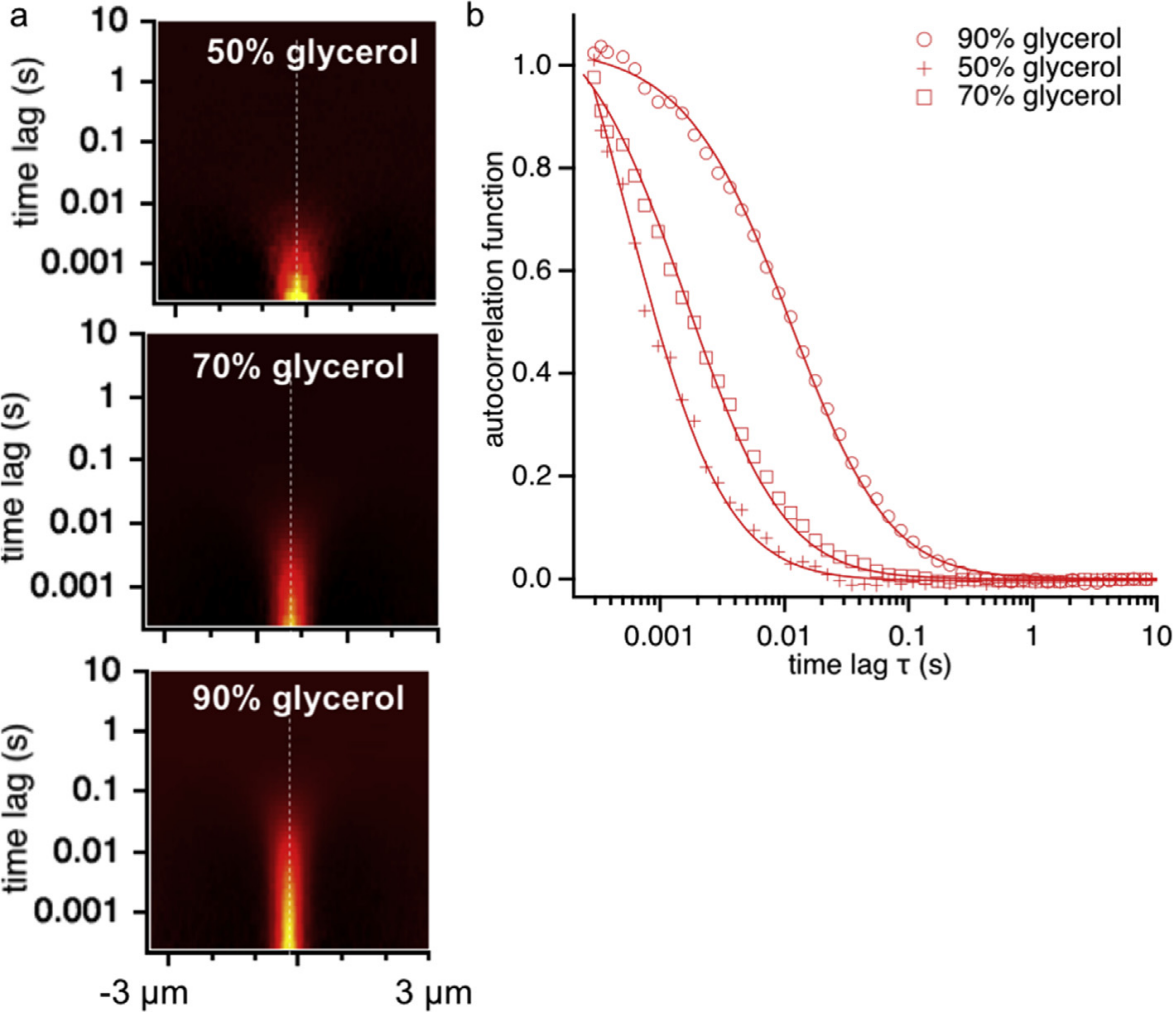
a) Representative images of the STICS functions for Alexa647 for increasing concentration of glycerol in the water-glycerol mixture. b) Autocorrelation function recovered from the vertical profiles highlighted in a, for spatial distance *x* = 0. Solid lines represent fits to the data according to eq. (1).

We enclose here first the measurements on three samples containing the dye Alexa 647, measured in water-glycerol mixtures of varying ratios. We report (Table 1) the measured diffusion coefficient in all three cases, together with the values expected based on the calculated viscosity of the water-glycerol mixtures, based on tabulated values [4]. Fig. 2a displays the representative image of the STICS functions calculated from the three datasets, whereas Fig. 2b is generated by taking the x = 0 section of the STICS functions in Fig. 2a (dashed lines). The data point markers are superposed to the fit (eq. (1)) used to extract the diffusion coefficients.

**Table 1:** Diffusion coefficients for the different samples investigated.

Glycerol, % Wt.	20 °C	25 °C	Measured
	D Theory^1^ (μm^2^/s)	D Theory (μm^2^/s)	D (RT) (μm^2^/s)
0	287 ± 9	330 ± 10	–
50%	48 ± 1	57 ± 2	53 ± 3
70%	12.8 ± 0.4	16.5 ± 0.5	17 ± 1
90%	1.31 ± 0 .04	1.85 ± 0.06	2.45 ± 0.05

**Fig. 3:**
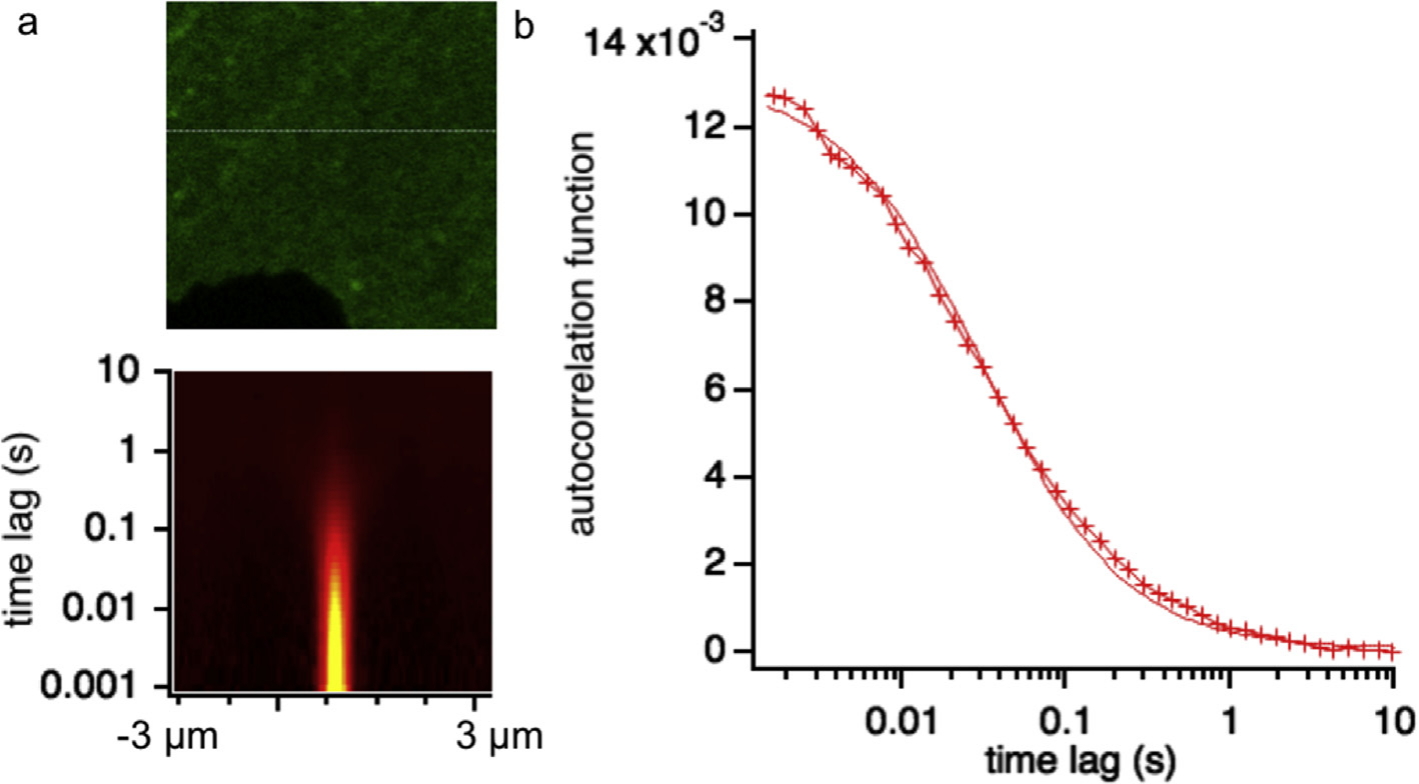
a) Confocal microscopy micrograph of H9c2 cell expressing a β2 Adrenergic Receptor, c-terminally fused to EGFP. b) STICS function calculated from the linescan collected along the line displayed in a. c) Autocorrelation function recovered from the vertical profiles highlighted in b (spatial distance *x* = 0). Solid line represents fit to the data according to eq. (2).

Fig. 3 illustrates the recovered autocorrelation function, and associated diffusion coefficient, calculated from a linescan performed on the plasma membrane of a living H9c2 cell expressing a prototypical G protein-coupled receptor, the β2 Adrenergic Receptor, c-terminally fused to EGFP. Cells were measured at 37 °C, 5% CO_2_. H9C2 cells are clonal cell lines derived from embryonic rat heart tissue and exhibit many of the properties of skeletal muscle.

## 2 Experimental design, materials, and methods

Linescan experiments were performed on a commercial confocal microscope (SP8-WLL, Leica Microsystems), equipped with a resonant scanner module (12 kHz) and a White Light Laser (WLL). A HC PLAPO CS2 40×1.3 NA Oil Immersion objective (Leica) was used in all the measurements. Alexa 647 (New England Biolabs) was excited at 633 nm with a laser power of 10%, corresponding to 6 μW total power at the sample. Approximately 2 × 10^6^ lines were collected. Measurements on the basolateral cell membrane were performed with the normal microscope galvanometer scanners at a speed of 1.8 kHz. EGFP was excited at 488 nm with a laser power of 5%, corresponding to 1 μW total power at the sample. Approximately 3 × 10^5^ lines were collected. Linescans were acquired 24 h after transfection. Zoom settings were chosen in order to achieve a pixel size of 50 nm. In both cases bleaching of the sample was minimal. Laser power was measured using a PM100A Power Meter (Thorlabs) using a S120VC (Thorlabs) Photodiode Power Sensor head.

Water-glycerol mixtures were prepared by weighting the glycerol (Merck Chemicals GmbH) and adding the appropriate volume of water and fluorescent compound. The solutions were extensively mixed by vortexing and were imaged in custom made imaging chambers: two stripes of parafilm (Bemis Company) were melted between a glass slide and a microscope coverslip (24 mm, #1, VWR), yielding in a chamber with a volume of approximately 50 μL. Dye concentration was measured by absorbance using a Spectrophotometer (Evolution 350, Agilent).

Cells were maintained at physiological conditions (37 °C, 5% CO_2_) using a sample incubator (Stage Top Chamber, OKOlab) mounted on the stage of the Leica SP8 confocal microscope.

H9c2(2-1) cells were obtained from ATCC® (CRL 1446™) and cultured as recommended by the seller. For imaging cells were grown in 8-well glass bottom μ-slides (Ibidi) and transfected using Lipofectamine 2000 transfection reagent (Thermofisher Scientific).

Linescan data were analyzed according to a previously described algorithm [2,5]. Briefly, the 2D autocorrelation function of the central portion (32 < x < 96 pixels) of the linescan is calculated (Fig. 1) and the profile at x = 0 used to extract the autocorrelation function. In the autocorrelation of data originating from the Leica SP8 microscope, the spatial autocorrelation of the linescan (line τ = 0) displays a sharp peak, possibly originating from electronic pixel to pixel correlations along the same line. This disappears as soon as τ > 0. Therefore, the first temporal time lag of the 2D correlation function is ignored by our subsequent analysis.

The autocorrelation function can then be fit with a standard 3D (eq. (1)) or 2D diffusion model (eq. (2)) in order to recover the diffusion coefficient of the fluorescent species. Here D is the diffusion coefficient, w_0_ the Point Spread Function in the xy plane (perpendicular to the optical axis of the objective) and w_z_ the beam waist along the direction of the optical axis. Our calibration, based on observing the profiles of fluorescence microspheres (Tetraspeck, Thermofisher Scientific) yields w_0_^633nm^ = 0.33 μm, w_z_^633nm^ = 1.12 μm, and w_0_^488nm^ = 0.28 μm, w_z_^488nm^ = 0.9 μm [6].

(1) $$G\left(x=0,\tau \right)=G\left(0\right)\frac{1}{\left(1+\frac{4D\tau }{w_{0}^{2}}\right)}\frac{1}{\sqrt{\left(1+\frac{4D\tau }{w_{z}^{2}}\right)}}$$

(2) $$G\left(x=0,\tau \right)=G\left(0\right)\frac{1}{\left(1+\frac{4D\tau }{w_{0}^{2}}\right)}$$

## References

[1] Ries J., Chiantia S., Schwille P. (2009). Accurate determination of membrane dynamics with line-scan FCS. Biophys. J..

[2] Di Rienzo C., Annibale P. (2016). Visualizing the molecular mode of motion from a correlative analysis of localization microscopy datasets. Opt. Lett..

[3] Hebert B., Costantino S., Wiseman P.W. (2005). Spatiotemporal image correlation spectroscopy (STICS) theory, verification, and application to protein velocity mapping in living CHO cells. Biophys. J..

[4] Dorsey N.E. (1940). s.

[5] Serfling R., Seidel L., Bock A., Lohse M.J., Annibale P., Coin I. (2019). Quantitative single-residue bioorthogonal labeling of G protein-coupled receptors in live cells. ACS Chem. Biol..

[6] Cole R.W., Jinadasa T., Brown C.M. (2011). Measuring and interpreting point spread functions to determine confocal microscope resolution and ensure quality control. Nat. Protoc..

